# Assessment of food insecurity and its associated factors among adult diabetic patients in Gambella town public hospital, South Western Ethiopia, 2023

**DOI:** 10.3389/fcdhc.2025.1493312

**Published:** 2025-09-08

**Authors:** Zeleke Girma, Mehari Teka, Direslgne Misker, Yilma Chisha, Mintesinot Melka Gujo, Endashew Shibru, Mamud Umer Wakeyo, Lidetu Timiketu, Temesgen Mohammed Toma, Yosef Haile

**Affiliations:** ^1^ South Ethiopia Region Health Bureau Public Health Institute, Jinka, Ethiopia; ^2^ Gambella Teachers Education and Health Science College, Gambella, Ethiopia; ^3^ Department of Public Health, Arba Minch University, Arba Minch, Ethiopia; ^4^ South Ethiopia Region Health Bureau, Jinka, Ethiopia; ^5^ Department of Epidemiology and Biostatistics, School of public health, Wolaita Sodo University, Wolaita Sodo, Ethiopia; ^6^ Arbaminch General Hospital, Monitoring & Evaluation, Arbaminch, Ethiopia

**Keywords:** food insecurity, diabetic patients, cross-sectional study design, Gambella town, Ethiopia

## Abstract

**Introduction:**

Food insecurity is a multidimensional issue that has been related with poor overall health, obesity and chronic diseases and not only related with increased prevalence of diabetes but also with increasing health care expenses. There is paucity of researches conducted to assess food insecurity and its associated factors among adult diabetic clients in Ethiopia. Hence this study was aimed to assess food insecurity and its associated factors among adult diabetic patients in Gambella town public hospitals.

**Methods:**

A facility based cross sectional study was conducted among adult diabetic clients in Gambella town public hospital from May 1 to June 30, 2023. A systematic sampling technique was used to select a sample of 412 patients. Data were collected by trained data collectors using structured questionnaires. Data were checked for its completeness and consistence then entered into Epidata 4.6 and transported to SPSS version 26 for analysis. Bivariate analysis was done to make variables candidate for multivariate analysis at p-value <0.25. In multivariate analysis AOR with 95% CI were used to declare factors associated with food insecurity at p-value <0.05.

**Results:**

The prevalence of food insecurity was found to be 59.5% (95% CI: 54.6%-64.3%). In multivariate analysis the variables age 18 to 24 years (AOR=0.093,95% CI:0.02-0.30), able to read and write (AOR=4.31, CI:1.246-11.250), employed (AOR=0.20; 95% CI: 0.063-0.63), low wealth status (AOR=3.02, CI:1.265-4.788) OR=2.46, CI:1.265-4.788), medium wealth status (AOR=1.88, CI:1.002-3.815). Family size (AOR=0.48; 95% CI: 0.27-0.87), and family history of diabetes (AOR=2.86, CI: 1.43- 5.72) were significantly associated with food insecurity. income and (AOR=1.88, CI:1.002-3.815)

**Conclusion and recommendations:**

The prevalence of food insecurity among adult diabetic patients was high. Therefore, health professionals should give emphasis to encourage self-management for those who have family history of diabetes in order to screen the clients at early before the occurrence of DM complication and health care expenditure.

## Introduction

Food insecurity refers to the limited and inconsistent availability, accessibility, utilization and stability of food. It is an important community health problem that remains mainly critical in place of chronic health conditions, like diabetes, that require dietary modification to manage (1).

Food insecurity has been associated with poor overall health, poor mental health, obesity and chronic diseases (2–4). In fact, adults who lack access to adequate food and nutrition are 2 to 3 times more likely to develop diabetes than those who do not. Food and nutrition insecurity are not only associated with increased prevalence of diabetes but also with increasing health care expenses. Diabetics with low FI and self-efficacy have more than twice health care utilization than food secure even after being stabilized in hospital, FI patients have double likelihood of being readmitted within 15 days of discharge and this is potentially harmful and can result in severe diabetes-related complications (5).

Diabetic patients often do not present to the hospital until they have severe complications related to previously undiagnosed diabetes, particularly those who do not have the economic means to see a physician regularly. FI is an important target for diabetes prevention and management since it is associated with poor glycemic control, higher rates of complications and hospitalization, and poor adherence to treatment. FI screening among individuals with diabetes could help healthcare workers to identify patients’ difficulties in adhering to treatment and dietary recommendations (6)

Globally, the prevalence of moderate and severe FI was predictable at 28.9%, with significant numbers of the people (2.33 billion) experiencing hunger in 2023. That means they did not have access to adequate food. It was also estimated that about 10.7% of the world’s population that means about 864 million people were food insecure at severe level. The prevalence of moderate or severe food insecurity in Africa was over twice as high as the global average in 2023 (58.0 percent), whereas the prevalence is closer to the global estimate in Asia, Latin America and the Caribbean, and Oceania (24.8, 28.2, and 26.8 percent, respectively) (7). According to the world food program (WFP) report 10.2 million people are severely food insecure including the 3 million people who are internally displaced in Ethiopia in 2025 (8). There are numerous studies that have described FI as an independent risk issue for poor intermediate health outcomes and may be associated with chronic disease in adults with low-income, including obesity, insulin resistance, T2DM, and cardiovascular diseases (9, 10). FI can compound diabetes management challenges by directly or indirectly influencing the three pillars of optimal glycemic control: nutrition therapy, physical activity, and self-management (11). Although genetic factors had a big influence on the development of diabetes and environmental factors also contributed to illness (12). FI is related with inadequate income as well as higher housing costs, higher rates of joblessness and nations with market taxes (13).

In Ethiopia; diabetic mellitus is a main community health problem in the modern era. Alongside more than half of diabetic patients not practicing the perceived healthy nutritive based approach, gathering with family and friends and eating out of home were the major reasons for not being in line with this (14). Food Security was essential in the managing of diabetic mellitus cases and its complications. Particularly, diets with low glycemic index, little total cholesterol reduce the risk of diabetic patients from developing complications. Adult diabetic patients should continue their food treatment throughout their whole course of diabetic care. There are three types of domains of food insecurity such as worry about not getting enough food, inadequate food quality and inadequate food quantity and it has been recognized as a theoretically modifiable risk factor that can be related with both the development of T2DM (15) and poor glycemic control (15).

Even if the previous research has identified a number of factors associated with both FI and diabetes, the factors that explain the relationship between FI and glycemic control remain unclear and there was also paucity of researches conducted in Ethiopia regarding to food insecurity and its associated factors among adult diabetic patients (15). The objective of this study was to assess food insecurity and its associated factors among adult diabetic patients in Gambella Town Public Hospitals, South Western Ethiopia. Addressing FI may help tackle the burgeoning challenge of diabetes in Ethiopia particularly in Gambella town.

## Methods

### Study design and period

A facility-based cross-sectional study was conducted from May 1 to June 30, 2023.

### Study area

**Figure 1:**
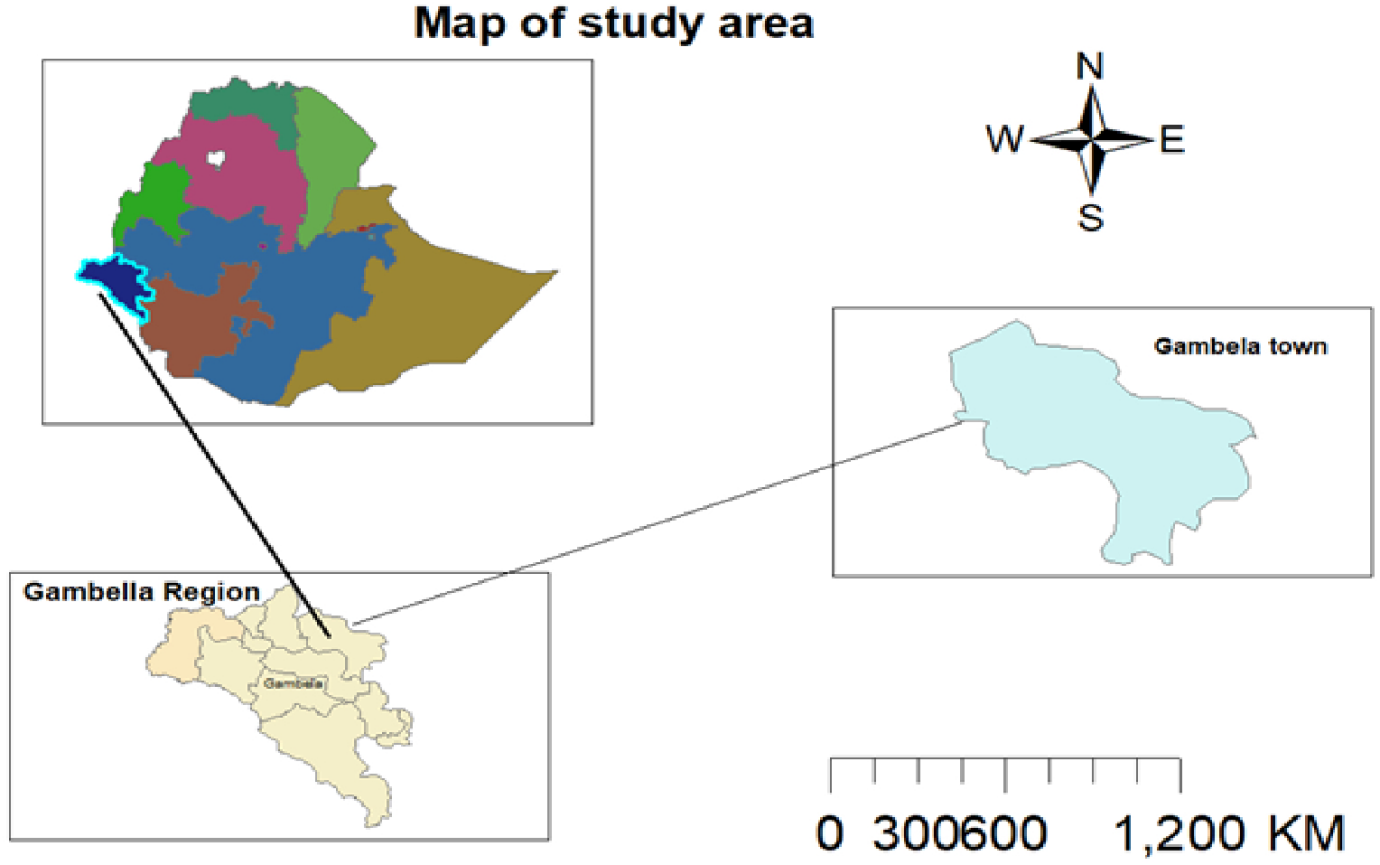
Map of Gambella town, March 2023.

The study took place in Gambella town’s public general and primary hospitals. Gambella town is located 768 kilometers southwest of Addis Ababa, the capital city of Ethiopia. The town is situated at the confluence of the Baro River and its tributary, the Jejebie. It has a latitude and longitude of 8°15’N 34°35’E and an elevation of 526 meters above sea level, featuring a hot climate. According to a 2023 report by the town health office, Gambella has a population of 67,451, comprising various ethnic groups, predominantly the Nuer, Agnuhak, and Majang. There are also settlers from other highlands of the country (**Figure 1**).

The study population consisted of adult diabetic patients attending DM clinics during the data collection period. Adult DM patients aged 18 and above visiting diabetic clinics during the study period were included whereas severely ill patients and pregnant mothers were excluded from the study.

### Sample size determination

The sample size was calculated using a single population proportion formula, assuming a 50.7% proportion of food insecurity (FI) among DM clients based on a previous study (15). With a 95% confidence level and a 5% margin of error, the calculated sample size was 384. Accounting for a 10% non-response rate, the final sample size was 422. The sample size for secondary objectives was lower, so the larger sample size was used.

### Sampling technique

The study involved two public hospitals having a total of 1350 DM clients: 900 from Gambella General Hospital and 450 from Gambella Primary Hospital. A systematic random sampling technique was used after proportional allocation based on client flow. Diabetic clinic services are provided five days a week. The sampling interval was calculated by dividing the average monthly flow of diabetic clients by the sample size. For Gambella General Hospital, K=900/281 = 3; for Gambella Primary Hospital, K=450/141 = 3. The initial number was randomly selected from one to three by lottery, resulting in two.

### Data collection technique and quality control

Data was collected through face-to-face interviews using structured questionnaires adapted from various literature sources. The questionnaire covered socio-economic, anthropometric and diabetic-related knowledge questions, diet, and the Household Food Insecurity Access Scale (HFIAS). Data collection was performed by four skilled diploma nurses and two BSc midwifery supervisors from Gambella town public hospitals. To maintain data quality, a two- day training session was conducted for data collectors and supervisors. The questionnaire was prepared in English, translated to Amharic, and back-translated to English to ensure consistency. The questionnaire was pretested on 5% of the sample size at a nearby health center, and data completeness was checked daily by the supervisor.

### Operational definitions for the study variables

Adequate glycemic control: When the FBS measurements 70 mg/dL - 126 mg/dL.

Inadequate glycemic control: When FBS measurements ≥126 mg/dL.

Diabetic knowledge: Patients who answered below the average of 23 DM knowledge assessment questions were labeled as having poor knowledge; those who scored above average were labeled as having good knowledge (15).

Food-secure: Q1a=0 or Q1a=1) and Q2 - Q9 = 0 (15)

Mild Food Insecurity: Q1a=2 or 3, or Q2a=1,2, or 3 or Q3a or Q4a=1 and Q5-Q9 = 0 (6)

Moderate Food Insecurity: Q3a or Q4a=2 or 3, or Q5a or Q6a=1 or 2, and Q7- Q9 = 0 (16)

Severe Food Insecurity: Q5a or Q6a=3, or Q7a, or Q8a, or Q9a=1,2 or 3 (17)

Wealth index: scores were given based on the number and kinds of consumer goods and housing characteristics. These scores were derived using principal component analysis and classified into low, middle and high wealth index (18).

### Data processing and analysis

After checking for completeness and consistency, data was cleaned, coded, and entered into Epidata version 4.6, then exported to SPSS version 26 for analysis. Descriptive statistics was used to show frequency distribution and principal component analysis (PCA) for wealth index was also performed. Bivariate analysis identified candidate variables for multivariate analysis at p-values <0.25. Adjusted Odds Ratios (AOR) with 95% Confidence Intervals (CI) and p-values <0.05 were used to identify statistically significant factors associated with food insecurity. Multicollinearity was checked using Variance Inflation Factor (VIF). Model goodness-of-fit was assessed using Hosmer-Lemeshow’s test, which yielded a result of 0.084, indicating a good fit.

### Ethical approval

ethical clearance was obtained from the Institutional Review Board of Arba Minch University College of Medicine and Health Science (Protocol No: MT1443/2023). Official permission was secured from Gambella town public hospitals. Participants were provided verbal consent before interviews, and confidentiality was maintained throughout the study. Participants were informed that refusal to consent or withdrawal from the study would not affect their access to healthcare.

## Results

### Socio-economic characteristics

In this study the overall response rate was found to be 412 (97.6%) among these 221 (53.6%) were females and the mean age ± SD was 43.1 ( ± 12.2) years and 287 (60.9%) respondents were urban dwellers. 296 (71.8%) of the respondents were married and 289 (70.1%) of the participants had <5 family sizes. Moreover, concerning occupation almost half of the respondents (51.2%) were housewives. Last but not least, one hundred forty-nine of the study participants (36.2%) were in the lowest wealth status (**Table 1**).

**Table 1:** Socio-economic characteristics of diabetic clients who had medical follow up in Gambella town public hospitals, Southwestern Ethiopia, 2023.

Variable	Category	Frequency	Percent
Sex	Male	191	46.4
	Female	221	53.6
Age	18-24	22	5.3
	25-34	87	21.1
	35-44	199	48.3
	45 & above	104	25.2
Residency	Urban	287	60.9
	Rural	125	30.3
Marital status	Single	46	11.2
	Married	296	71.8
	Divorced	45	10.9
	Widowed	25	6.1
Family size	<5	289	70.1
	≤5	123	29.9
Educational status	Unable to read & write	57	13.8
	Able to read & write	41	10
	Primary/Secondary	103	25
	Diploma and above	211	51.2
Wealth index	Low	149	36.2
	Medium	119	28.9
	High	144	35

### Health and anthropometric characteristics of diabetic patients

In this study, 350 (85%) of the respondents had a disease duration of <5 years and 153 (37.1%) of the respondents had DM related comorbidities. Of the DM patients, 331(80.3%) were taking oral treatment and 64 (15.5%) of them were using insulin medications. Two hundred ninety-two (70.9%) of the respondents did not get education related to DM and 336 (81.5%) of the respondents reported that they don’t know the presences of DM association. 322 (78.2%) of the respondents had faced inadequate glycemic control. Moreover, in this study 275 (66.7%) of the respondents had inadequate knowledge related to DM. (**Table 2**).

**Table 2:** Health and anthropometric characteristics of diabetic clients who had medical follow up in Gambella town public hospitals, Southwestern Ethiopia, 2023.

Variable	Category	Frequency	Percent
DM Duration	≤5 year	350	85
	6-10	52	12.4
	11-20	11	2.7
Family history of DM	yes	94	22.8
	No	318	77.2
DM education	yes	120	29.1
	No	292	70.9
Source of DM education	Media	57	13.8
	Health worker	58	14.1
	Other*	6	1.5
	None	291	70.6
Member of DM association	Yes	76	18.4
	No/Don’t know	336	81.5
Fast Blood Sugar	Adequate GC	90	21.8
	Inadequate GC	322	78.2
Waist hip ratio	Low risk	77	18.7
	Medium risk	238	57.7
	High risk	97	23.6
DM knowledge	Good	137	33.3
	Poor	275	66.7

Other* = Family, Friend, Neighbor, and Non-diabetic client.

### Diet related characteristics

In this study it was reported that two hundred fifty-three (61.4%) of the study participants consumes food outside their home and 247 (60%) of them reported as their consumption was ≤ 4 days per week. 318 (77.8%) the respondents did not fast before diabetic diagnosis and for one hundred twenty (29.1%) of the participants the most fasting hours was 7-10. In addition to this, 373 (90.5%) of the respondents worried about the high cost of food (**Table 3**).

**Table 3:** Diet related characteristics of diabetic clients who had medical follow up in Gambella town public hospitals, Southwestern Ethiopia, 2023.

Variable	Category	Frequency	Percent
Eating out of home	Yes	153	61.4
	No	159	38.6
Number of days for eating out of home per week	1-4	247	60
	5-7	4	1
	None	161	39.1
Nutrition education	Yes	162	39.3
	No	250	60.7
Source of nutrition education	Health worker	73	17.7
	Media	80	19.4
	Friend	7	1.7
	None	272	61.2
Worry about high cost of foods	Yes	373	90.5
	No	39	9.5
Fasting before DM	Yes	159	38.6
	No	253	61.4
Hours fasting	1-6	39	9.5
	7-10	120	29.1
	None	253	61.4

### Magnitude of food insecurity among adult diabetic patients

**Figure 2:**
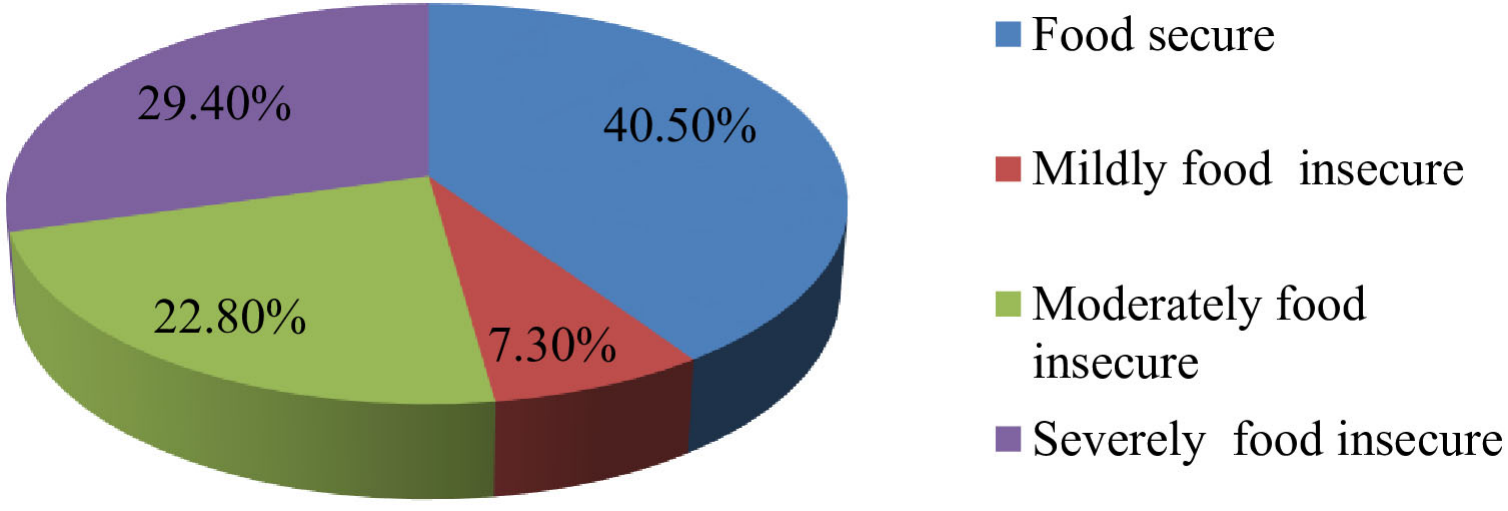
Levels of food insecurity among adult diabetic’s wgo had medical follow up in Gambella town in public hospitals, Southwestern Ethiopia, 2023.

The overall magnitude of food insecurity in this study was found to be 59.5% (54.6% - 64.3%). The level of FI classified as Food Secure (40.5%), mildly FI (7.3%), moderately FI (22.8%), and severely FI (29.4%), (**Figure 2**). In this study concerning the domain of Food Insecurity it was reported that two hundred forty five (59.5%) of the participants worry about not getting enough food, 236(57.5%) unable to eat preferred foods, 263(63.8%) has limited variety of foods, 213(51.7%) feed unwanted food, 266(64.6%) eat a smaller meal, 235(57%) eat fewer meals in a day, 68(16.5%) has no any kind food in house, 139(26.1%) go to sleep at night hungry, and 71(17.2%) go hungry a day and night (**Table 4**).

**Table 4:** Magnitude of food insecurity among adult diabetic clients who had medical follow up in Gambella town public hospitals, Southwestern Ethiopia, 2023.

Question of occurrence	Yes (%)	No (%)	Frequency of occurrence Question N (%)			
			0	1	2	3
Worry about food	245 (59.5)	167 (40.5)	167 (40.5)	87 (21.1)	137 (33.3)	21 (5.1)
Unable to eat preferred foods	236 (57.5)	106 (42.5)	106 (42.5)	121 (29.37)	102 (24.76)	13 (3.15)
Limited variety of foods	263 (63.8)	118 (28.6)	118 (28.6)	143 (34.7)	109 (26.5)	11 (2.7)
Feeding unwanted food	213 (51.7)	199 (48.3)	199 (48.3)	115 (27.9)	94 (22.8)	4 (1.0)
Eat a smaller meal	266 (64.6)	146 (35.4)	146 (35.4)	*167 (40.5)*	*90 (21.8)*	*9 (2.2)*
Eat fewer meals in a day	235 (57)	177 (43)	177 (43)	155 (37.6)	74 (18)	6 (1.5)
No any kind food in house	68 (16.5)	344 (83.5)	344 (83.5)	38 (9.2)	26 (6.3)	3 (.7)
Go to sleep at night hungry	139 (26.1)	273 (66.3)	273 (66.3)	120 (29.1)	16 (3.9)	3 (.7)
Go hungry a day and night	71 (17.2)	341 (82.8)	341 (82.8)	68 (16.5)	14 (3.4)	82 (19.9)

Response options for frequency-of-occurrence question of HFIAS labeled as: (1= rarely, 2= sometimes, 3= often).

### Associated factors with FI among adult diabetic patients

Based on bivariate analysis variables like sex, age, residence, marital status, family size, educational status, occupational status, wealth status, duration of DM, comorbidity, DM medication, family history of DM, education on DM, Source of DM education, member of DM association, fasting before DM diagnosis, WHR, knowledge on DM, nutrition education, and worry about high costs were candidate variables at p<0.25.

In multivariate analysis, age, able to read and write, occupational status of house wife, low and medium in wealth status, and having family history of DM were significantly associated with FI at p value<0.05. The odds of food insecurity were reduced by 90.7% (AOR=0.093, CI:0.02-0.30), 78% (AOR= 0.22, CI:0.086-0.465) and 63% (AOR= 0.37, CI: 0.19-0.76) among age group 18-24, 25-34, and 35-44, respectively than age group 44 and above. The odds of FI was 4.31 times higher among able to read and write as compared to diploma and above (AOR=4.31, CI:1.246-11.250). The odd of FI was reduced by 80% (AOR=0.20; 95% CI: 0.063-0.63), and 88% (AOR=0.12, CI: 0.039- 0.43) among employed and merchant study participants respectively as compared to those who are housewife. The odds of FI was 3.02 and 1.88 times higher among low- and medium-income respondents than high income (AOR=3.02, CI:1.265-4.788) and (AOR=1.88, CI:1.002-3.815) respectively. Moreover, the odds of FI was about 2.8 times higher among participants having family history of diabetic compared to their counter parts (AOR=2.86, CI: 1.43- 5.72) (**Table 5**). Furthermore, the odds of FI was reduced by 52% (AOR=0.48; 95% CI: 0.27-0.87) among study participants with less than 5 family members as compared to those who have more than or equal to 5 family members.

**Table 5:** Factors associated with food insecurity among adult diabetic clients who had medical follow up in Gambella town public hospitals, Southwestern Ethiopia, 2023.

Variable	Category	Food insecurity status		COR (95% CI)	AOR (95% CI)	P value
		Food insecure	Food secure			
Age in years	18-24	9 (3.7)	13 (7.8)	0.145 (0.05-0.39)	0.093 (0.02-0.30)	0.001
	25-34	35 (14.5)	52 (31.1)	0.141 (0.07-0.27)	0.22 (0.086-0.46)	0.001
	35-44	115 (46.9)	84 (50.3)	0.287 (0.16-0.51)	0.37 (0.19-0.76)	0.007
	45and above	86 (35.1)	18 (10.8)	1	1	
Educational level	Unable to read and write	45 (18.4)	12 (7.2)	4.162 (2.08-8.31)	1.089 (0.40-2.95)	0.866
	Able to read and write	34 (13.9)	7 (4.2)	5.391 (2.28-12.71)	3.574 (1.25-10.25)	0.018
	1ry&2ry school	66 (26.9)	37 (22.2)	1.980 (1.22-3.22)	1.552 (0.76-3.16)	0.226
	Diploma and above	100 (40.8)	111 (66.5)	1	1	
Occupation status	Employed	115 (46.9)	116 (69.5)	0.29 (0.083-0.69)	0.20 (0.063-0.63)	0.010
	Merchant	33 (13.5)	23 (13.8)	1.447 (0.80-2.62)	0.12 (0.04- 0.43)	0.001
	Unemployed	61 (24.9)	23 (13.8)	2.675 (1.55-4.61)	2.100 (0.95-4.63)	0.127
	House wife	36 (14.7)	5 (3)	1	1	
Wealth Index	Low income	110 (44.9)	39 (23.4)	3.241 (1.98-5.29)	3.02 (1.26-4.78)	0.008
	Middle income	68 (27.8)	51 (35.5)	1.532 (0.94-2.49)	1.88 (1.00-3.81)	0.049
	High income	67 (27.3)	77 (46.1)	1	1	
Family history of DM	Yes	74 (30.2)	20 (12)	3.181 (1.85-5.46)	2.86 (1.43- 5.72)	0.003
	No	171 (69.8)	147 (88)	1	1	
Nutrition education	No	110 (44.9)	52 (31.1)	1	1	
	Yes	135 (55.1)	115 (68.9)	0.555 (0.37-0.83)	0.353 (0.20- 0.61)	0.051
Duration of diabetes diagnosis	≤ year	211 (86.1)	139 (83.2)	1	1	
	6-10	30 (12.2)	21 (12.6)	0.941 (0.52-1.71)	0.594 (0.26-1.33)	0.204
	11-20	4 (1.6)	7 (4.2)	0.376 (0.11-1.31)	0.170 (0.03-0.99)	0.059
Family size	<5	153 (62.5)	136 (81.4)	3.181 (1.85-5.46)	0.48 (0.27-0.87)	0.001
	≥5	92 (37.5)	31 (18.6)	1	1	

## Discussion

This study aimed to assess the magnitude and associated factors of food insecurity among diabetic patients in Gambella town public hospitals, Southwest Ethiopia. The results revealed that 59.5% (54.6% - 64.3%) of adult diabetic patients experienced FI. This finding is consistent with studies conducted in South Africa (63.6%) (19) Iran (59.7%) (20) the United States (56%) (20). However, it is higher than the prevalence reported in Addis Ababa (50.7%) (20) and Canada (19.5%**).** This may be potentially due to differences in poverty and income. That means people who live in Addis Ababa have high income rates as compared to Gambella region in general. In addition, there is a limited employment opportunity in Gambella region as compared to Addis Ababa that may limits the accessibility of sufficient nutritious food. Conversely, it is lower than the prevalence reported in Burkina Faso (71%) (21) and Jordan (78%) (22) which may be attributed to variations in geographical location, socio-economic status, study settings, and sample sizes.

Younger age groups were less likely to experience food insecurity compared to older age groups, aligning with findings from the National Health Interview Survey 2016 (23) and a similar study in Nigeria (24). This could be due to younger diabetic patients have higher chance of employment and get income providing a high chance of getting adequate food among younger diabetic patients. In addition, younger patients likely to have health insurance coverage because of higher employment that increases the chance of obtaining health services.

Participants who could read and write were 3.57 times more likely to be food insecure compared to those with diploma or higher. This is consistent with studies conducted at Nekemte Referral Hospital (16) and in Southwest Iran (25), suggesting that education creates self-awareness regarding a healthy and quality diet. Higher education also leads to higher employment or better job opportunities and better salaries this may lower the food insecurity among educated as compared to uneducated diabetic patients. Moreover, more educated diabetic patients may have good social networks that may increases access to information resources and in generally the support.

Housewives were more likely to experience food insecurity compared to employed individuals. This finding aligns with studies in South Africa (26) and Europe (27), indicating that housewives may rely on cash crops rather than edible crops and housewife entirely depend on their husbands for financial support. This financial dependence aggravates the food insecurity level at household level. Housewife may have limited control on the resources that are available in their home this can leads to difficulty for them to obtain nutritious food by them. housewives may have limited level of education as compared to government employee and merchant this may limit access to basic information about healthy diets, and available resources.

This study also showed that the odds of food insecurity were higher among low- and medium-income households than high income households respectively, which is consistent with studies in Canada (27), California and Chicago (28). This might be due to those who have low or middle income may face to buy necessary food items this may leads to food insecurity at their household. Often, they face difficult to make choices among different competing needs for instance between buying food items, for rent, transportation, health services and education.

Moreover, participants with a family history of DM were more likely to experience food insecurity compared to those without. This aligns with WHO findings indicating that offspring of individuals with T2DM have a higher risk of food insecurity (29). Study participants with family history of DM may have other members in their house who are diabetic which may lead them to financial catastrophe because it makes them to pay high costs for health services/drugs, monitoring materials like glucose meters and for diabetic related complications and at the end this leads to them to have less money for food. In addition, restricted type of foods is recommended for diabetic patients these recommended food types are costly as compared to other types of foods therefore this may affect food insecurity at the household level.

Furthermore, the odds of FI was reduced among study participants who have less than 5 family members as compared to those who have more than or equal to 5 family members. This might be because family’s with small number of members have low dependency ratio this reduces the burden among productive group in the family. They may also pay low cost/low expenses for food, education, transport, rent, and health care if they have family size. Having fewer family size could leads to greater per capital income because there are fewer family members who need support in the households this may decreases food insecurity at the household level.

### Limitations of the study

The HFIAS questions relied on the respondents’ memory of events over the previous four weeks, which could introduce response and recall bias. Additionally, the cross-sectional design cannot establish a cause-and-effect relationship. Moreover, we have included so many independent variables in this study to identify the associated factors this may leads to in the interpretation and communication of the finding. For this reason, we have tried to focus on the most important variables in detail.

## Conclusion

The proportion of food insecurity among adult diabetic patients in Gambella town public hospitals is high. Predictors of food insecurity include age, rural residence, literacy, housewife occupation, low and medium wealth status, non-membership in DM associations, nutritional education, longer DM duration, and family history of DM. Health professionals should emphasize self-management for individuals with a family history of DM to prevent complications and reduce healthcare costs. Encouraging participation in DM associations can enhance access to nutritional education and counseling. Moreover, higher officials should work to improve household income alongside other stakeholders.

## Data Availability

The raw data supporting the conclusions of this article will be made available by the authors, without undue reservation.

## References

[1] Reeder N Tapanee P Persell A Tolar-Peterson T. Food insecurity, depression, and race: Correlations observed among college students at a university in the Southeastern United States. Int. J. Environ. Res. Public Health. (2020) 17:8268., PMID: 33182386 10.3390/ijerph17218268 PMC7664923

[2] Haering SA Syed SB. Community food security in United States cities: A survey of the relevant scientific literature. Center for a Livable Future. Baltimore, USA: Johns Hopkins Bloomberg School of Public Health (2009). Available at: https://wwwjhsphedu/sebin/s/c/FS_Literature%20Bookletpdf.

[3] Gordon B. The impact of food insecurity on glycemic control among individuals with type 2 diabetes. BioMed. (2022) 2:170–80. doi: 10.3390/biomed2020016

[4] Lee JS Frongillo EA Jr. Nutritional and health consequences are associated with food insecurity among US elderly persons. J. Nutr. (2001) 131:1503–9. 10.1093/jn/131.5.1503 11340107

[5] Krupsky KL Sliwa S Seligman H Brown AD Liese AD Demissie Z. Adolescent health risk behaviors, adverse experiences, and self-reported hunger: Analysis of 10 states from the 2019 youth risk behavior surveys. J. hunger Environ. Nutr. (2024) 19:523–39. doi: 10.1080/19320248.2022.2088263, PMID: 38954493 PMC10300635

[6] Zeleke Negera G Charles Epiphanio D. Prevalence and predictors of nonadherence to diet and physical activity recommendations among type 2 diabetes patients in southwest Ethiopia: A cross-sectional study. Int. J. endocrinology. (2020) 2020:1512376., PMID: 32190048 10.1155/2020/1512376 PMC7064825

[7] WHO FIUW. The State of Food Security and Nutrition in the World 2024 Financing to end hunger, food insecurity and malnutrition in all its forms. (2024).

[8] WFP. Saving Lives Changing Lives Annual Country Report 2024 Ethiopia. (2025).

[9] Gearhardt A N Davis C Kuschner R D. Brownell K. The addiction potential of hyperpalatable foods. Curr. Drug Abuse Rev. (2011) 4:140–5. doi: 10.2174/1874473711104030140, PMID: 21999688

[10] Organization WH. The state of food security and nutrition in the world 2019: safeguarding against economic slowdowns and downturns. Food & Agriculture Org (2019).

[11] Nsimbo K Erumeda N Pretorius D. Food insecurity and its impact on glycaemic control in diabetic patients attending Jabulani Dumani community health centre, Gauteng province, South Africa. Afr. J. Primary Health Care Family Med. (2021) 13:1–6. doi: 10.4102/phcfm.v13i1.2906, PMID: 34082551 PMC8182565

[12] Mohammed MA Sharew NT. Adherence to dietary recommendation and associated factors among diabetic patients in Ethiopian teaching hospitals. Pan Afr. Med. J. (2019) 33. doi: 10.11604/pamj.2019.33.260.14463, PMID: 31692826 PMC6814932

[13] Cheng S Kamano J Kirui N Manuthu E Buckwalter V Ouma K. Prevalence of food insecurity in patients with diabetes in western Kenya. Diabetic Med. (2013) 30:e215–e22. doi: 10.1111/dme.2013.30.issue-6, PMID: 23506405

[14] Kushel MB Gupta R Gee L Haas JS. Housing instability and food insecurity as barriers to health care among low-income Americans. J. Gen. Internal Med. (2006) 21:71–7., PMID: 16423128 10.1111/j.1525-1497.2005.00278.x PMC1484604

[15] Berkowitz SA Baggett TP Wexler DJ Huskey KW Wee CC. Food insecurity and metabolic control among US adults with diabetes. Diabetes Care. (2013) 36:3093–9. doi: 10.2337/dc13-0570, PMID: 23757436 PMC3781549

[16] Tezera R Sahile Z Yilma D Misganaw E Amare E Haidar J. Food security status of patients with type 2 diabetes and their adherence to dietary counselling from selected hospitals in Addis Ababa, Ethiopia: A cross-sectional study. PloS One. (2022) 17:e0265523. doi: 10.1371/journal.pone.0265523, PMID: 35421127 PMC9009691

[17] Mukai N Doi Y Ninomiya T Hata J Hirakawa Y Fukuhara M. Cut-off values of fasting and post-load plasma glucose and HbA1c for predicting Type 2 diabetes in community-dwelling Japanese subjects: the Hisayama Study. Diabetes Med. (2012). doi: 10.1111/j.1464-5491.2011.03378.x, PMID: 21726278

[18] WHO. Noncommunicable Disease Surveillance, Monitoring and Reporting. WHO (2024). Available at: https://www.who.int/teams/noncommunicable-diseases/surveillance/systems-tools/steps.

[19] Kabakov E Norymberg C Osher E Koffler M Tordjman K Greenman Y. Prevalence of hypertension in type 2 diabetes mellitus: impact of the tightening definition of high blood pressure and association with confounding risk factors. J. cardiometabolic syndrome. (2006) 1:95–101. doi: 10.1111/j.1559-4564.2006.05513.x, PMID: 17679829

[20] Kharisma V Abe N. Food insecurity and associated socioeconomic factors: Application of Rasch and binary logistic models with household survey data in three megacities in Indonesia. Soc. Indic. Res. (2020) 148:655–79. doi: 10.1007/s11205-019-02210-z

[21] Coates J Swindale A Bilinsky P. Household Food Insecurity Access Scale (HFIAS) for measurement of food access: indicator guide: version 3. (2007).

[22] Thomas MK Lammert LJ Beverly EA. Food insecurity and its impact on body weight, type 2 diabetes, cardiovascular disease, and mental health. Curr. Cardiovasc. Risk Rep. (2021) 15:1–9., PMID: 34249217 10.1007/s12170-021-00679-3 PMC8255162

[23] Tabrizi JS Nikniaz L Sadeghi-Bazargani H Farahbakhsh M Nikniaz Z. Socio-demographic determinants of household food insecurity among Iranian: a population-based study from northwest of Iran. Iranian J. Public Health. (2018) 47:893., PMID: 30087876 PMC6077642

[24] Shalowitz M Eng J McKinney C Krohn J Lapin B Wang C. Food security is related to adult type 2 diabetes control over time in a United States safety net primary care clinic population. Nutr. diabetes. (2017) 7:e277–e. doi: 10.1038/nutd.2017.18, PMID: 28504709 PMC5518800

[25] Janzadeh H Mozaffari-Khosravi H Javadi M. The association of food insecurity, inflammation, and several socioeconomic factors with type 2 diabetes: A case-control study. J. Nutr. Food security. (2020) 5:38–46.

[26] Mendoza JA Haaland W D'Agostino RB Martini L Pihoker C Frongillo EA. Food insecurity is associated with high risk glycemic control and higher health care utilization among youth and young adults with type 1 diabetes. Diabetes Res. Clin. practice. (2018) 138:128–37. doi: 10.1016/j.diabres.2018.01.035, PMID: 29427695 PMC5910177

[27] Bawadi HA Ammari F Abu-Jamous D Khader YS Sa B Tayyem RF. Food insecurity is related to glycemic control deterioration in patients with type 2 diabetes. Clin. Nutr. (2012) 31:250–4., PMID: 22119231 10.1016/j.clnu.2011.09.014

[28] Fekadu G Bula K Bayisa G Turi E Tolossa T Kasaye HK. Challenges and factors associated with poor glycemic control among type 2 diabetes mellitus patients at Nekemte Referral Hospital, Western Ethiopia. J. Multidiscip. healthcare. (2019), 963–74. doi: 10.2147/JMDH.S232691, PMID: 31819470 PMC6878927

[29] Mohammad A Majid K Hosssen H. The comparison of food insecurity between patients with type 2 diabetes mellitus and non-diabetic controls referred to rural health centers in abadan. J. NutritionFasting Health. (2019) 7:18–25.

